# Successful surgical treatment for squamous cell carcinoma arising from hidradenitis suppurativa: A case report and literature review

**DOI:** 10.1097/MD.0000000000005857

**Published:** 2017-01-20

**Authors:** Cheng Huang, Zhichao Lai, Mu He, Biyun Zhai, Liangrui Zhou, Xiao Long

**Affiliations:** a Division of Plastic and Reconstructive Surgery, Peking Union Medical College Hospital, Beijing, P.R. China; b Department of Medicine, the University of Melbourne, Parkville, Victoria, Australia; c Department of Pathology, Peking Union Medical College Hospital, Beijing, P.R. China.

**Keywords:** hidradenitis suppurativa, squamous cell carcinoma, surgery

## Abstract

**Rationale:**

Hidradenitis suppurativa (HS) is a disabling inflammatory disease mainly affecting apocrine glands. Marjolin ulcer (MU) is a term used to describe a rare type of squamous cell carcinoma (SCC) arising within sites of chronic wounds or preexisting scars. Chronic HS may result in a rare type of SCC, MU, which has a poor prognosis due to its high metastatic rate.

**Concerns of the patient:**

Here we reported a 60-year-old male who developed SCC on the right buttock after suffering from HS for 15 years.

**Interventions:**

Radical resection with clear margin was performed, after which topical negative pressure (TNP) was applied followed by split-thickness skin grafting.

**Outcomes:**

In a 1-year follow-up, there was no recurrence of malignancy.

**Lessons:**

Cases reported in English literature since 1991 were reviewed to get a general grasp of status quo. The authors conclude that chronic HS lesion especially in the gluteal region should be cautiously observed. Once tumor arisen from HS lesion, immediate radical excision should be performed. With assured clear margin, TNP could be chosen to offer a favorable environment for the survival of skin grafting.

## 1 Introduction

Hidradenitis suppurativa (HS), also known as acne inversa and Verneuil disease, was first described by Velpeau in 1832. The prevalence of HS was estimated approximately 0.053% to 4.1% worldwide. Since the missed diagnosis rate of HS is high, the morbidity may be higher than that.^[1,2]^ HS is a chronic, cicatricial disease mainly affecting apocrine-bearing areas in young and middle-aged adults. Histologic findings recognized it as a disorder of the follicular epithelium.^[3]^ It often afflicts patients for many years with pain, malodor, and disfigurement. HS is brought to the attention of general or plastic surgeons only after the dissatisfaction of multiple long-term trials of conservative therapy. Among those consequences, Marjolin ulcer (MU) has the poorest prognosis due to its high metastatic rate. MU is a term used to describe a rare type of squamous cell carcinoma (SCC) arising within sites of chronic wounds or preexisting scars.^[4]^ Due to the low incidence, comprehensive review of the MU caused by HS is strongly needed. Here we presented the clinical and pathological characteristics as well as the treatment of MU. In addition, we also reviewed the recent reports on SCC arising from HS since 1991.

### 1.1 Consent

Written informed consent was obtained from the patient for publication of this case report and any accompanying images. A copy of the written consent is available for review by the Editor of this journal.

## 2 Case report

**Figure 1:**
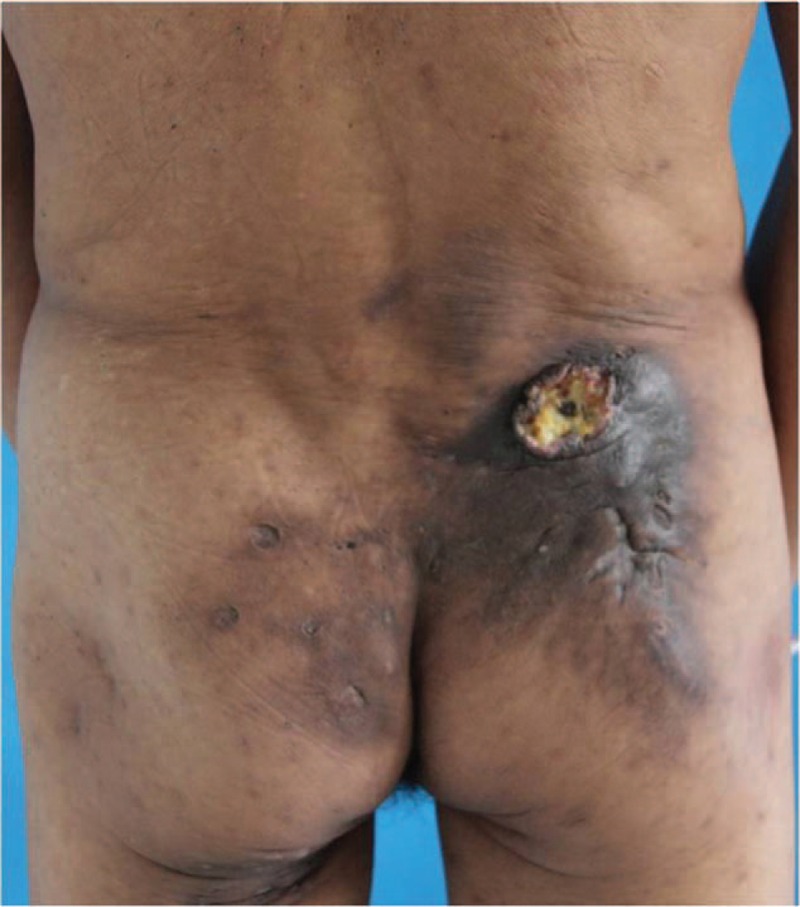
Mass (5 cm × 5 cm) with volcanic-like ulcer in the right buttock.

**Figure 2:**
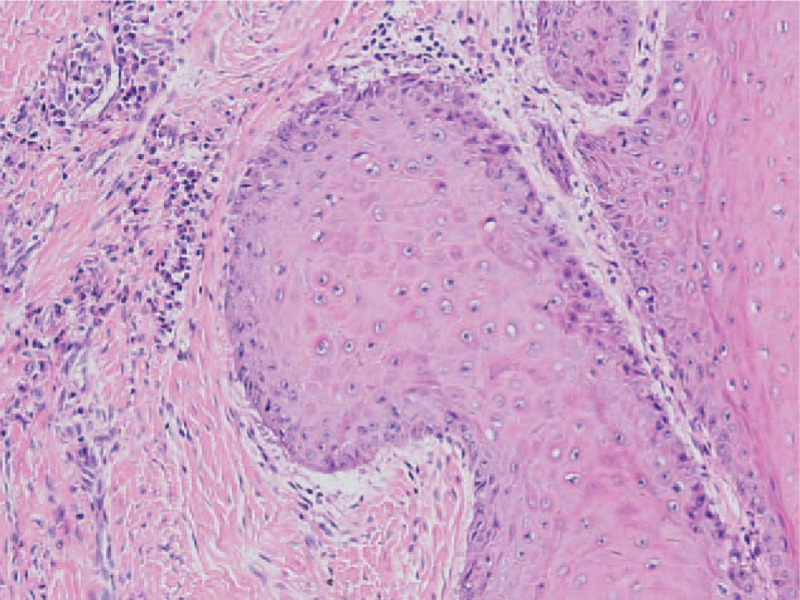
Second biopsy of the mass showing well-differentiated SCC. Hematoxylin and eosin ×100.

A 60-year-old male was suffering from repeated painful furuncles in the right buttock for 15 years. The lesion was mainly on the buttocks and perineal region accompanied by hypertrophy of the surrounding tissue. During this period, the patient had received incision and furuncle drainage for several times, however, the lesions were frequently recurrent. By May 2014, he developed an ulcerative tumor in the right buttock which forced him to consult further treatment. He was diagnosis of HS by dermatologist and then administrated with oral antibiotics. His medical history was unremarkable except for a 40-year smoking habit. Physical examination revealed a 5 cm × 5 cm mass in the right buttock with crateriform ulcer and stinky purulence on the top (Fig. 1). Besides, the surrounding tissue was hyperpigmented with tenderness. Pathological biopsy was performed twice right after diagnosis. The first examination revealed slight skin hyperkeratosis, irregular acanthosis, pseudoepitheliomatous hyperplasia (PEH) and infiltration of a few chronic inflammatory cell surrounding the perivasculatures in the superficial dermis. Second biopsy demonstrated verrucous carcinoma (a highly differentiated SCC) (Fig. 2), immunohistochemistry results showed negative human papillomavirus (HPV), p16 expression and high Ki67. Meanwhile, bacterial cultivation of the drained nodules revealed proteus mirabilis. Before admission, he was treated with chemotherapy for a week. Nevertheless, he terminated the therapy because of unbearable pain.

**Figure 3:**
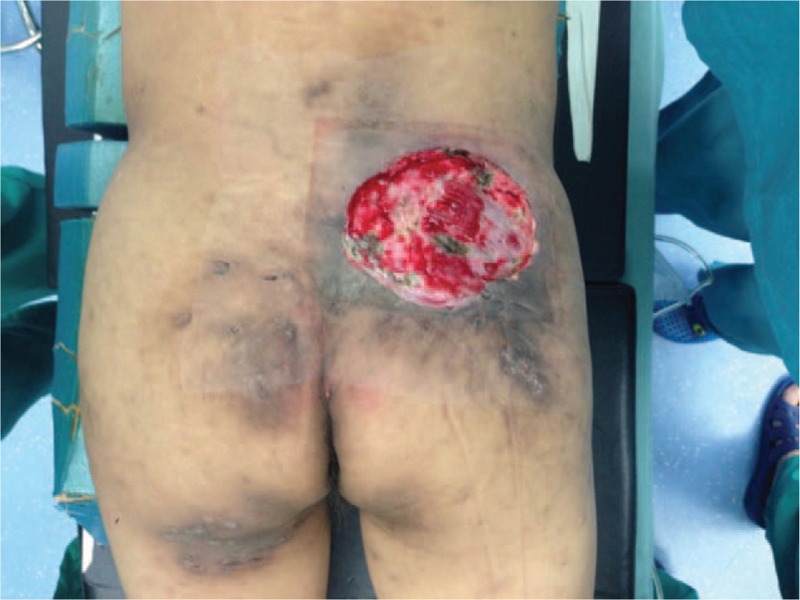
Defection left after radical excision of the lesion in the right buttock.

**Figure 4:**
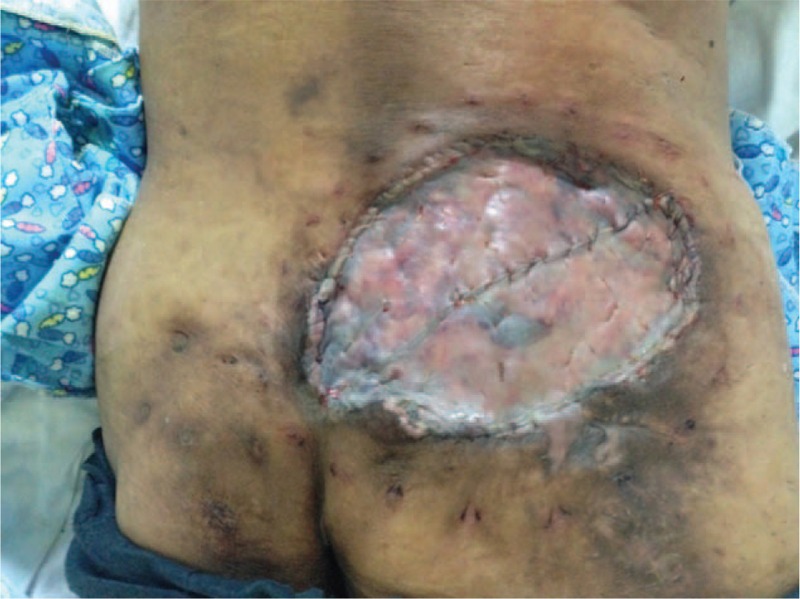
Wound covered by split-thickness skin grafting healed well after applying topical negative pressure for a week.

In July 4, 2014, radical resection till deep fascia and 3 cm free margin was performed under local anesthesia. During operation frozen pathologies of resection margin in the direction of 3, 6, 9, 12 o’clock showed tumor free (Fig. 3). Topical negative pressure (TNP) treatment was applied consecutively for a week and fresh granulation tissues was formation. Later, reconstruction with split-thickness skin grafting from lateral thigh was conducted, the skin grafting healed primarily under careful observation (Fig. 4). A year later, there is no evidence of tumor recurrence in this patient.

## 3 Discussion

### 3.1 Methodology of review

**Figure 5:**
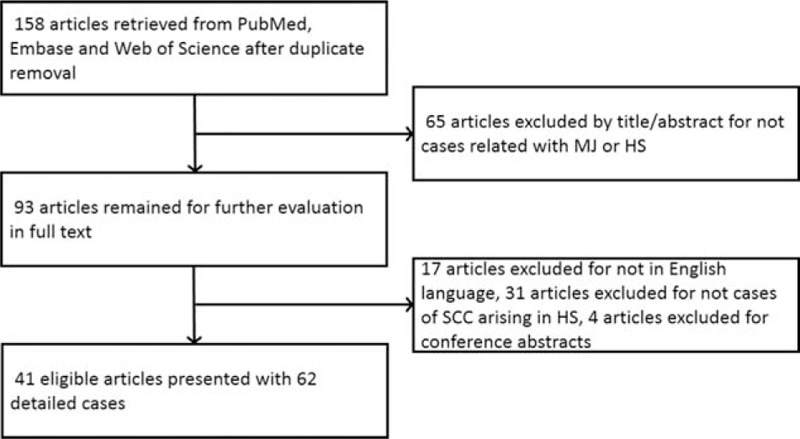
Flow chart for screening eligible articles.

SCC arising from HS is also named as MU, which was characterized by aggressiveness and ulcerating. The incidence of MU arising from long-standing HS varies from 1% to 3.2%.^[5]^ Transformation from HS to SCC may be explained by chronic irritation and infection, which lead to proliferative epidermal changes and increased rate of spontaneous mutations. By far, HS complicating SCC was rarely reported despite a low occurrence rate of HS. To get a general grasp, we carried out a comprehensive search of “PubMed,” “Embase,” and “Web of science” using the following keywords: “hidradenitis suppurativa,” “acne inversa,” “Verneuil disease,” “follicular occlusion triad,” and “squamous cell carcinoma,” “Marjolin Ulcer” in English literature from January 1991 until now. Cases published before 1991 were excluded because they were out of date and had been reviewed by Williams et al.^[6]^ The flow chart of searching strategy is demonstrated in Fig. 5.

### 3.2 Epidemiologic characteristics

A total of 62 cases were reported from 41 separate articles. The overall information is demonstrated in Table 1  . HS was diagnosed with an average age of 27.49 ± 10.16 years old ranging from 14 to 53 years old, which indicated that a younger HS onset may get a high malignant tendency as normally the onset age was in the second or third decade of life. The average diagnosis age of SCC was 54.12 ± 10.14 years old, while the average latency period of SCC developed from HS was 27.13 ± 9.93 years. Nevertheless, Chang et al^[2]^ reported an acute malignancy transformation from HS lesion despite a more than 30 years latency period. Yu et al^[7]^ divided the latency period into preulceration and postulceration periods, pointing out that duration of ulceration played an important role in malignant transformation. Therefore cautious surveillance and more aggressive treatment should be executed in ulcerated patients. Even though female had a high prevalence of HS, those who suffered from MU are practically male with a ratio of 6.75:1. The most predisposing site of Marjolin ulcer (MJ) included buttocks and perianal region, existing in most cases and sometimes affecting sacrum, groin, thighs, and vulva region. Regional factor may also play a role in SCC formation as it seldom affected the axillary area. One possible explanation may be that male tends to have a high morbidity in anogenital or perineal region while female in axillary.^[8]^ Concomitant diseases accompanied with these cases included hypercalcemia,^[5,9]^ follicular occlusion triad,^[10]^ Crohn disease,^[11]^ osteomyelitis,^[12]^ spina bifida,^[13]^ polyneuropathy,^[14]^ etc.

**Table 1:**
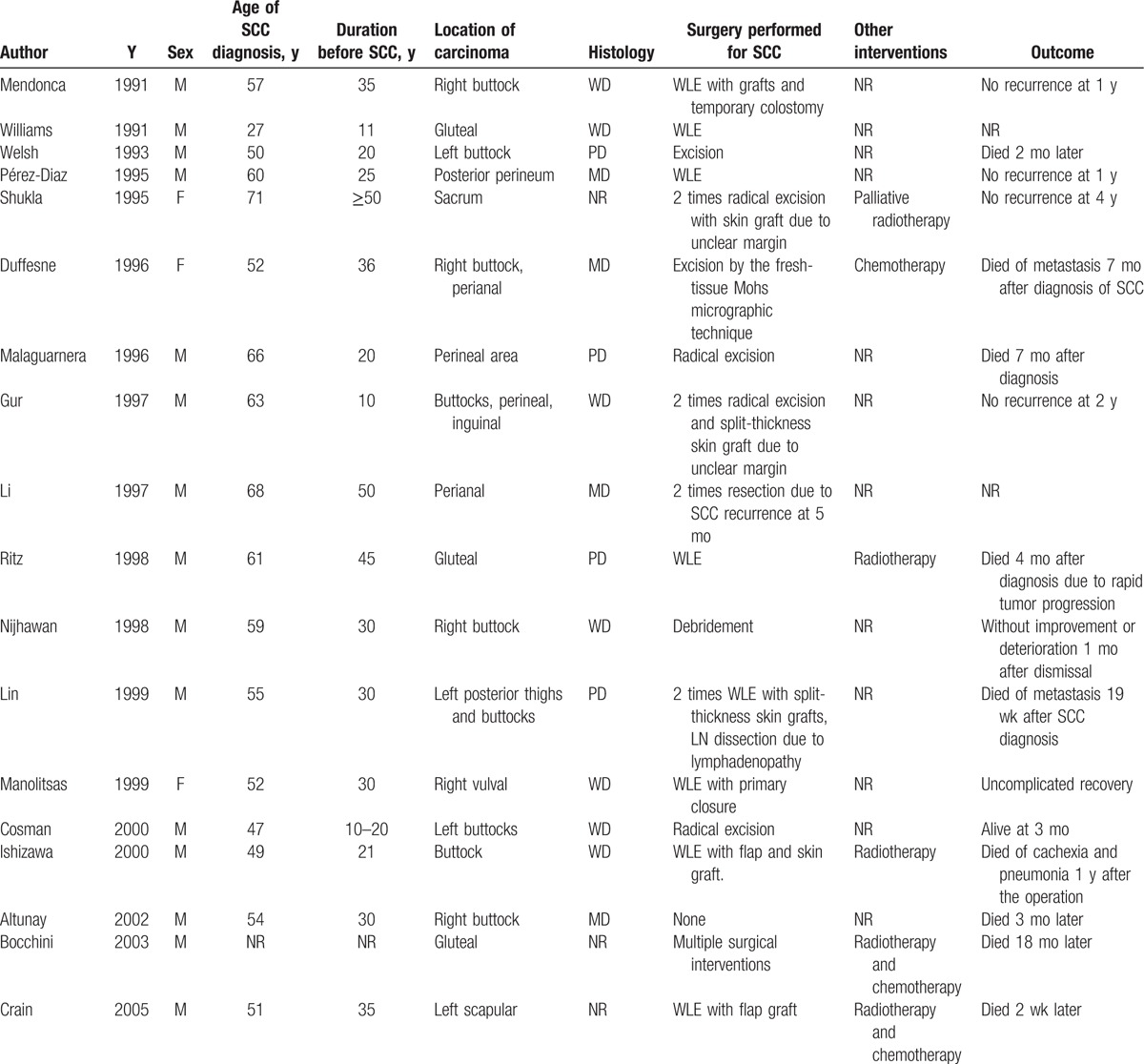
Overall information of patients with squamous cell carcinoma arising from hidradenitis suppurativa since 1991.

**Table 1 (Continued):**
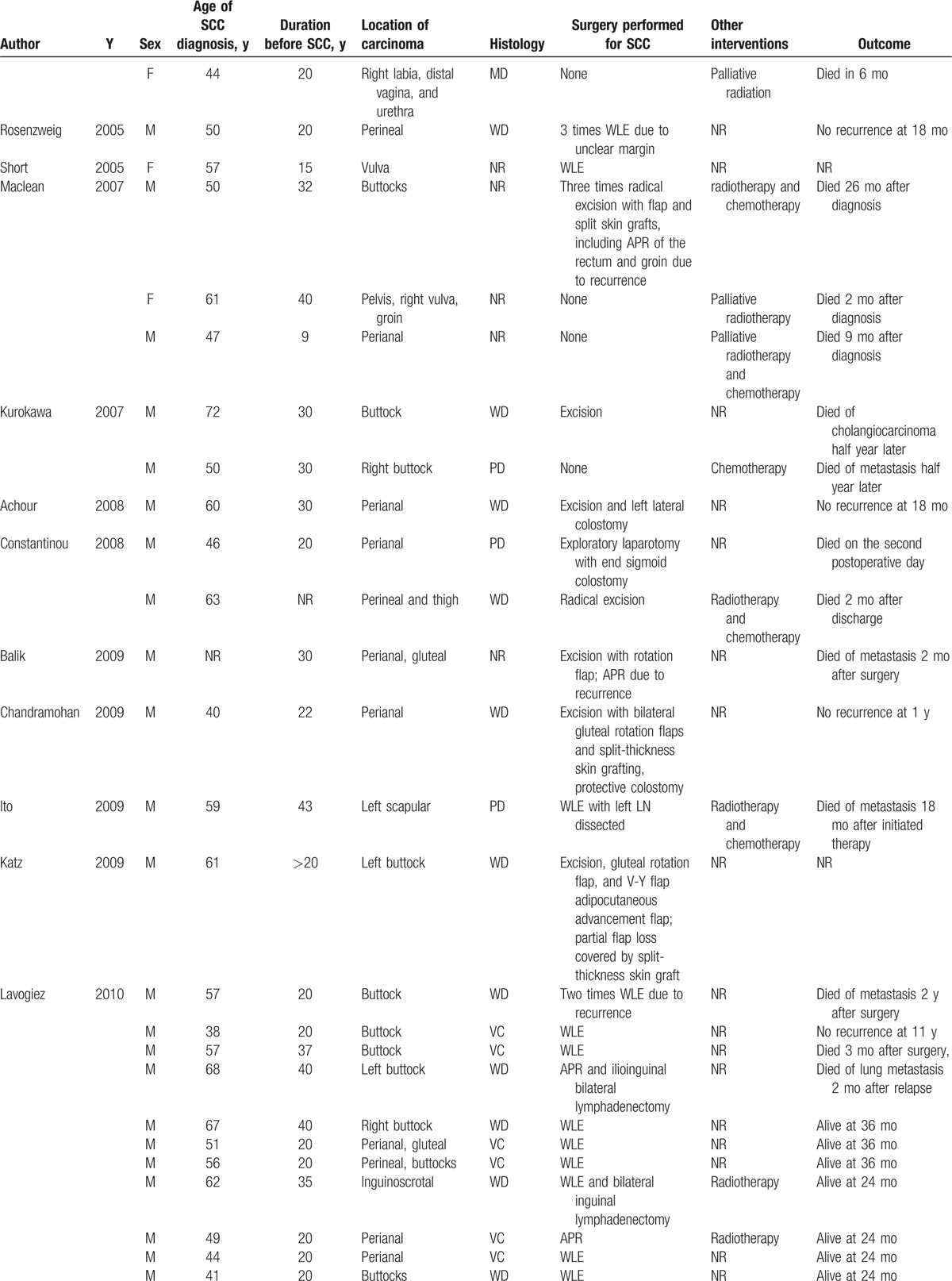
Overall information of patients with squamous cell carcinoma arising from hidradenitis suppurativa since 1991.

**Table 1 (Continued):**
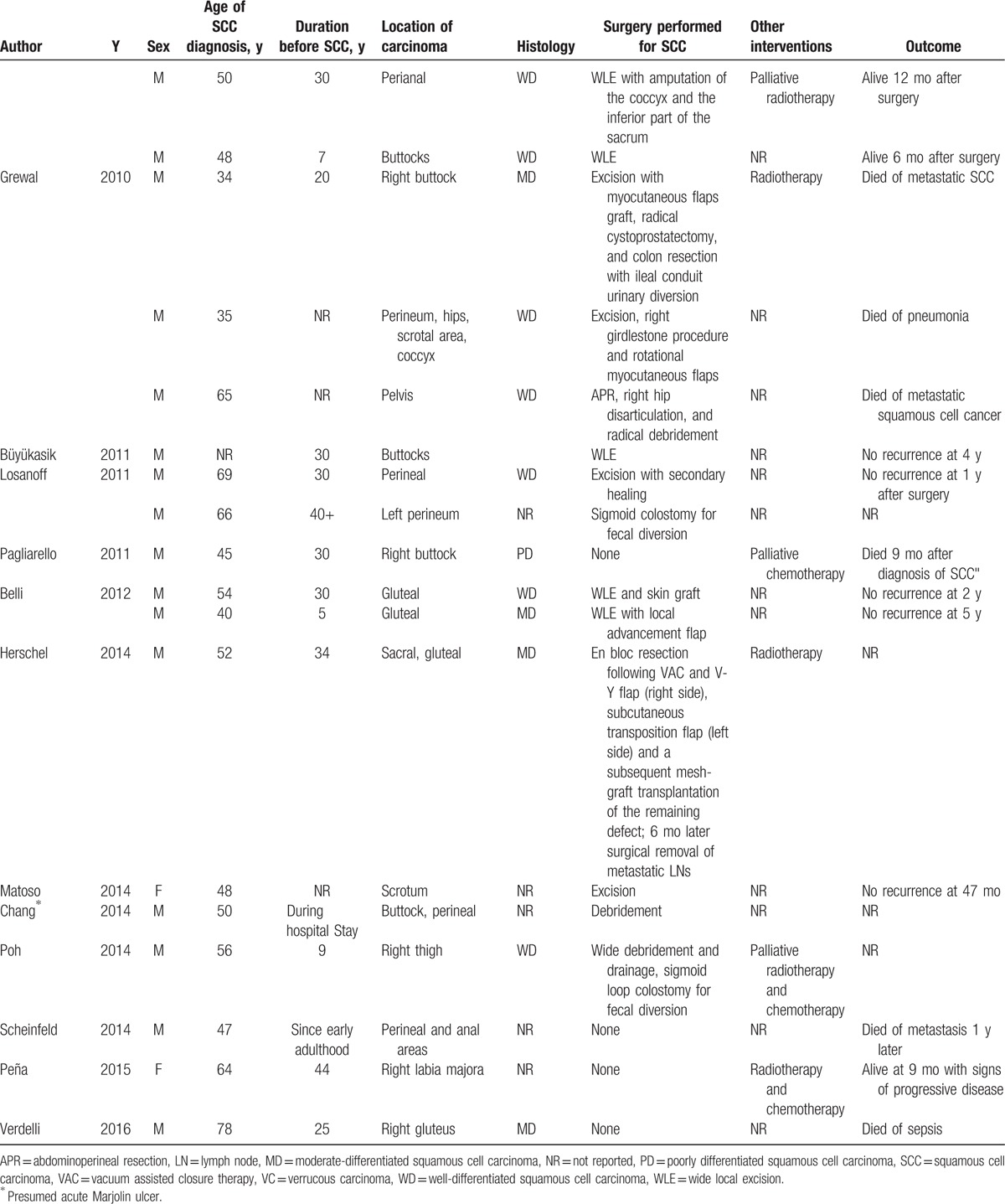
Overall information of patients with squamous cell carcinoma arising from hidradenitis suppurativa since 1991.

### 3.3 Pathology and prognosis

Pathology was recorded according to Broder classification. Even though well and moderately differentiated SCC accounted for the vast majority of MU (85%), nearly half patients died within 2 years. Possible reasons were listed as follow: Identification of malignant transformation on the background of chronic skin inflammation is relatively difficult; presence of sinus tract allows easy spread of cancer; lesion biopsy sometimes presents false negative results just like the case we presented. Thus, it tends to have a long journey from the onset of MU to final treatment. Besides, HPV infection was assumed to play a carcinogenic role in SCC arising in HS.^[15]^ It was concluded that positive p16/HPV expression with high Ki67 was associated with basaloid/warty morphology of SCC, while negative p16/HPV expression with low Ki67 was associated with usual morphology.^[16]^ However, our case (verrucous carcinoma) was inconsistent with the former conclusion since negative HPV/p16 expression with high Ki67 was demonstrated.

### 3.4 Treatment recommendation

Based on American Joint Committee on Cancer Guidelines, primary surgical excision was recommended for invasive cutaneous SCC. Radiation therapy was typically reserved for patients who are unable to undergo surgical excision while chemotherapy has not been proved to be effective. According to our review, 53 out of 62 cases received surgical intervention. To avoid the existence of small focus of tumor, meticulous excision with clear margins of tumor biopsy are recommended.^[17]^ Lavogiez et al^[15]^ suggested a minimum margin of 2 cm. However, few articles described their methods in detail on wound coverage. In our experience, split-thickness skin grafting is recommended compared with flap as it facilitate the early detection of tumor recurrence. TNP have the properties of increasing wound oxygen tension, antibacterium, promoting granulation formation as well as comforting patients.^[18,19]^ Considering the recurring nature of HS and secondary infection in buttock lesion, we used TNP to provide a favorable environment for the survival of skin grafting. Clear margin should be fully assured otherwise it may facilitate tumor dissemination. In most circumstances, HS does not involve the endoanal mucosa, so colostomy is not a regular procedure except when the patients has fecal incontinence or extensive, complicated perianal disease.^[6]^

## 4 Conclusion

HS may get malignant transformation under chronic inflammatory irritation. Cautious surveillance and active intervention should be taken especially in the gluteal involvement subtype of ulcerating patients.^[20]^ Once SCC was found in HS lesion, radical excision with extended region and clear margins should be performed timely to avoid tumor metastasis. By virtue of TNP, split-thickness skin grafting could be survived even under chronic inflammatory environment. Regular follow-up should be taken in case of tumor recurrence.
